# Large-scale analysis of B-cell epitopes of envelope: Implications for Zika vaccine and immunotherapeutic development

**DOI:** 10.12688/f1000research.16454.2

**Published:** 2019-06-27

**Authors:** Iman Almansour, Rahaf Alfares, Halah Aljofi

**Affiliations:** 1 Epidemic Diseases Department-Institute for Research and Medical Consultations (IRMC), Imam Abdulrahman Bin Faisal University, Dammam, Eastern Region, P.O.Box 1982, Dammam 31441, Saudi Arabia

**Keywords:** Antibody, Bioinformatics, Envelope, Epitope, Homology Modeling, Neutralizing Antibodies, Vaccine, Zika

## Abstract

**Background: **Cases of the re-emergence of Zika virus in 2015 were associated with severe neurologic complications, including Gillien-Barre syndrome in adults and congenital Zika syndrome in newborns. The major structural determinant of immunity to the Zika virus is the E protein. Although B-cell epitopes of Zika E protein were recently identified, data regarding epitope variations among Zika strains in pre-epidemic and epidemic periods are lacking.

**Methods: **Here, we conducted systematic bioinformatics analyses of Zika strains isolated between 1968 and 2017. Multiple sequence alignment of E protein as well as B-cell epitopes annotations were performed. In addition, homology-based approach was utilized to construct three-dimensional structures of monomeric E glycoproteins to annotate epitope variations. Lastly, prediction of of *N*-glycosylation patterns and prediction of protein stability upon mutations were also investigated.

**Results:** Our analyses indicates that epitopes recognized by human mAbs ZIKV-117, ZIKV-15, and ZIKV-19 were highly conserved, suggesting as attractive targets for the development of vaccines and immunotherapeutics directed against diverse Zika strains. In addition, the epitope recognized by ZIKV-E-2A10G6 mAb derived from immunized mice was mostly conserved across Zika strains.

**Conclusions:** Our data provide new insights regarding antigenic similarities between Zika strains circulating worldwide. These data are essential for understanding the impact of evolution on antigenic cross-reactivity between Zika lineages and strains. Further *in-vitro* analyses are needed to determine how mutationsat predefined epitopes could impact the development of vaccines that can effectively neutralize Zika viruses.

## Introduction

Zika is a positive-sense, enveloped, RNA virus of the *Flaviviridae* family ^1^, which also includes dengue virus, West Nile virus (WNV), Japanese encephalitis virus (JEV), tick-borne encephalitis virus (TBEV), and yellow fever virus (YFV) ^2^. Zika was originally discovered in a rhesus monkey in 1947 in Uganda ^3^, and the first case of spread to humans was reported in 1952 ^4^. Since that time, the virus has spread globally, with Zika outbreaks reported in Micronesia in 2007 and in the Pacific islands in 2013–2014 ^5, 6^. A recent outbreak that began in Brazil in 2015 that eventually spread to countries in North America and the Caribbean ^7, 8^.

The Zika virus genome encodes three structural proteins (capsid [C], premembrane [PrM], and envelope [E]) and seven non-structural proteins (NS1, NS2A, NS2B, NS3, NS4A, NS4B, and NS5) ^2^. Similar to other flaviviruses, the structural proteins and viral genome form virions that assembles as immature particles at the endoplasmic reticulum of infected cells ^9^. The immature particles are composed of 60 PrM/E protein heterodimers that protrude from the viral surface ^10^. In the Golgi apparatus, PrM is cleaved by furin-like protease to produce mature M protein and Pr protein product ^2^. After maturation, PrM and E are released and 90 E protein homodimers rearrange in a herringbone-like array forming mature Zika virus ^11, 12^.

The E protein is the major surface glycoprotein of flaviviruses and plays an essential role in virus attachment and fusion. Each E protein monomer consists of three domains: DI, DII, and DIII ^13^, which undergo major rearrangements during the virus maturation cycle ^14, 15^. DI is a central beta-barrel domain; DII is a finger-like dimerization domain; and DIII is an immunoglobulin-like domain ^10, 11^. DI, which connects DII to DIII, is essential for the conformational changes required for viral entry into cells ^16^. DII contains a fusion loop (FL) that interacts with the endosomal membrane, whereas DIII contains the receptor-binding site and is thus essential for attachment of virus particles to the host cell ^15, 16^. DIII also plays an essential role in mediating the fusion of virus particles with the endosomal membrane after endocytosis ^17^.

The global spread of Zika virus in conjunction with the neurologic consequences of infection have increased the urgency of efforts to develop Zika vaccines and immunotherapeutics. Humoral immunity is the major source of host protection against flaviviruses, in which neutralizing antibodies play an important role in virus clearance ^18^. Antibodies generated against the E protein have been shown to block the entry of viruses into host cells ^19^. Previous attempts to map the antigenic epitopes of the Zika E protein utilized antibodies specific for other flaviviruses, such as dengue virus ^20– 22^. Recently, several B-cell antigenic epitopes within an individual E domain were identified in studies of antibodies isolated from Zika-virus infected patients ^23– 25^ and Zika-vaccinated mice ^13, 26, 27^.

Although antigenic epitopes of the E protein have been characterized based on maps prepared using Zika-specific antibodies, no systematic analyses of the specificity of Zika monoclonal antibodies (mAbs) for available Zika E protein sequences have been conducted. Such data are particularly important, as RNA viruses exhibit high mutation rates and can generate mutations that enable them to evade the host immune system. Importantly, the structural stability of E protein is a key factor for antibody binding. The amino acids substitutions and subsequent effects on structural stability and antibody binding can be performed by a homology based *in-silico* approach of the three-dimensional (3D) structure of E proteins. Hence, analyzing sequence data to annotate mutations at key residues and subsequent prediction of the effect of those mutations on protein structure stability is essential. Therefore, identifying novel amino acids mutations that are likely to contribute in the immune evasion is important.

In the present study, therefore, we extracted all of the available E protein sequences for Zika isolates obtained from 1968 to 2017 and constructed three-dimensional (3D) structures of E proteins from various Zika strains using homology modeling. We also investigated the patterns and conservation of E protein B-cell epitopes and assessed their structural stability upon mutation.

## Methods

### Data selection

Complete Zika polypeptide sequences for isolates identified between 1968 and 2017 were obtained from the National Center for Biotechnology Information (NCBI) Zika resource ^28^. A total of 409 complete polypeptide sequences were retrieved, and duplicate sequences were removed. Multiple sequence alignment was performed using MUSCLE in the Geneious tool, version 11.0, and the E protein region was extracted ^29^. Lastly, MUSCLE alignment was performed for E protein sequences and duplicate E sequences were subsequently removed.

### Phylogenetic analysis

A phylogenetic tree for all unique Zika virus E protein sequences was constructed using the maximum-likelihood method with the PhyML tool, version 3.0 ^30^, with 100 bootstrap replications. The tree applied an LG substitutional model to determine the divergence of E protein sequences. Lastly, the phylogenetic tree was edited using the Figtree tool, version 1.4.3.

### Homology modeling

The SWISS model ^31^ server was used to generate 3D structures of the E proteins of Zika isolates identified in 1968, 2007, 2013, 2015, and 2016. Chain A of the E protein structure (PDB: 5GZN) was used as a template for homology modeling. The best homology model was selected based on global model quality estimate (GMQE) and Qmean statistics. Each homologous 3D structure was evaluated using Ramachandran plots prepared with PROCHECK ^32^. Hydrogen bonds were added using molprobity ^33^. Each model was subjected to energy minimization using the ModRefiner server described by Xu and Zhang ^34^.

### Mapping of antigenic epitopes

E protein-specific antigenic epitopes of monoclonal antibodies (mAbs) isolated from Zika-virus infected humans and Zika immunized mice were retrieved from the Immune Epitope Database (IEDB) ^35^, which is a free resource funded by the National Institute of Allergy and Infectious Diseases devoted to disseminating antigenic epitope data. Linear and conformational B-cell epitopes with positive major histocompatibility complex ligands were selected. B-cell epitope regions mapped with B-cell receptor (BCR)-positive neutralizing antibodies were also selected. Epitopes that mapped with screening peptides and did not elicit an immune response were removed. A total of 7 human and 10 mouse (from mice immunized with E protein) B-cell epitopes were identified. The identified epitopes were annotated against aligned E sequences as well as the 3D structures of monomeric E proteins using the Chimera tool ^36^. Potential sites of *N*-glycosylation were predicted using NetNglycan 1.0 server ^37^. Potential *N*-glycosylation sites were defined by the sequence Asp/X/Ser/Thr, where X represents any amino acid except Pro. The default threshold of >0.5 was used as predictor of *N*-glycosylated residue.

### Analysis of mutations on E protein stability

The effect of mutations on the stability of E protein was predicted using the mutation cutoff scanning matrix (mCSM) ^38^, site-directed mutator (SDM) ^39^, DUET ^40^, and I-Mutant 2.0 ^41^ tools. The mCSM is a machine-learning algorithm based on a 3D physiochemical environment, and the data are summarized as a graphical signature. The SDM is a statistical potential energy function based on the propensity of amino acids in wild-type and mutant proteins to assume folded and unfolded conformations. DUET is an integrated computational approach that utilizes both SDM and mCSM to predict the effect of non-synonymous single-nucleotide polymorphisms on protein stability. Lastly, the I-Mutant webserver is a neural network-based tool for predicting mutation-associated free energy changes. The I-Mutant2.0 tool enables prediction of free energy changes under differing conditions of pH, temperature, neighboring residues, and solvent accessibility. For predicting protein stability, pH 7.0 and temperature at 25 C were applied.

## Results

### Strain frequencies

Antigenic variations among Zika strains were examined by first obtaining the complete sequences of Zika polypeptides from the NCBI Zika resource ^19^. A total of 409 Zika polypeptide sequences were retrieved. Identical polypeptide sequences were removed, resulting in a final total of 257 sequences. Sequences were aligned by MUSCLE using Geneious software, E protein sequences were extracted, and duplicate E protein sequences were removed, resulting in a total of 75 unique sequences ( Table 1). Of note, the majority of the 75 unique E protein sequences were represented strains isolated in 2015 and 2016. Sequences from isolates collected in 2017 did not harbor any unique mutations in comparison to sequences from isolates collected in previous years; thus, 2017 sequences were removed after duplicate E protein sequences were removed.

**Table 1.** List of unique E protein sequences and their frequency.

Number	Accession	Sequence Description	Frequency	N-Glycosylation
**1**	AMR68906	|Homo sapiens|Nigeria|09/09/1968		-
**2**	ACD75819	|Homo sapiens|Micronesia|01/06/2007		154 NDTG
**3**	AMR39834	|Homo sapiens|Cambodia|2010	2	154 NDTG
**4**	AMD61711	|Homo sapiens|Philippines|09/05/2012		154 NDTG
**5**	ANO46307	|Homo sapiens|French Polynesia|11/2013		154 NDTG
**6**	ANO46309	|Homo sapiens|French Polynesia|01/2014		154 NDTG
**7**	AMD61710	|Homo sapiens|Thailand|19/07/2014		154 NDTG
**8**	AMK49165	|Homo sapiens|Brazil|2015		154 NDTG
**9**	AMK49164	|Homo sapiens|Brazil|2015		154 NDTG
**10**	AOC50654	|Homo sapiens|Honduras|06/01/2015		154 NDTG
**11**	AMX81917	|Homo sapiens|Thailand|16/01/2015		154 NDTG
**12**	ASB32509	|Homo sapiens|Brazil|13/05/2015	4	154 NDTG
**13**	ALX35659	|Homo sapiens|Suriname|02/10/2015		154 NDTG
**14**	AMC13913	|Homo sapiens|Guatemala|01/11/2015		154 NDTG
**15**	AMD16557	|Homo sapiens|Brazil|30/11/2015		154 NDTG
**16**	ASU55392	|Homo sapiens|Colombia|12/2015		154 NDTG
**17**	ASU55393	|Homo sapiens|Colombia|12/2015		154 NDTG
**18**	ASU55394	|Homo sapiens|Colombia|12/2015		154 NDTG
**19**	ASU55403	|Homo sapiens|Colombia|12/2015		154 NDTG
**20**	ASU55399	|Homo sapiens|Colombia|12/2015		154 NDTG
**21**	ASU55411	|Homo sapiens|Colombia|12/2015		154 NDTG
**22**	ASU55407	|Homo sapiens|Colombia|12/2015	3	154 NDTG
**23**	ASU55396	|Homo sapiens|Colombia|12/2015		154 NDTG
**24**	ASU55398	|Homo sapiens|Colombia|12/2015		154 NDTG
**25**	ASU55409	|Homo sapiens|Colombia|12/2015		154 NDTG
**26**	AMQ48982	|Homo sapiens|Brazil|29/01/2016		154 NDTG
**27**	AMQ48986	|Homo sapiens|USA|02/02/2016		154 NDTG
**28**	AMK79469	|Homo sapiens|China|06/02/2016		154 NDTG
**29**	APC60216	|Homo sapiens|Mexico|03/03/2016		154 NDTG
**30**	AOY08538	|Homo sapiens|Brazil|21/03/2016		154 NDTG
**31**	AOX49265	|Homo sapiens|Italy|04/2016	2	154 NDTG
**32**	AQS26698	|Homo sapiens|South Korea|04/2016		-
**33**	AOY08534	|Homo sapiens|Brazil|05/04/2016	167	154 NDTG
**34**	ARB07987	|Homo sapiens|Dominican Republic|07/04/2016 Republic|07/04/20160Republic|2016/04/07		154 NDTG
**35**	ARB07941	|Homo sapiens|Brazil|14/04/2016		-
**36**	ARB07968	|Homo sapiens|Brazil|15/04/2016		154 NDTG
**37**	ARB07988	|Homo sapiens|Dominican Republic|18/04/2016 Republic|2016/04/18		154 NDTG
**38**	AQS26816	|Homo sapiens|Brazil|24/04/2016		154 NDTG
**39**	APG56499	|Homo sapiens|Taiwan|05/2016		154 NDTG
**40**	ATG29278	|Homo sapiens|Honduras|10/05/2016		154 NDTG
**41**	ARB07930	|Homo sapiens|Honduras|13/05/2016		154 NDTG
**42**	ATG29285	|Homo sapiens|Mexico|17/05/2016		154 NDTG
**43**	ART29823	|Homo sapiens|Russia|31/05/2016		154 NDTG
**44**	ARB07996	|Homo sapiens|DominicanRepublic|06/06/2016 r Republic04/07/4 Republic|2016/06/06		154 NDTG
**45**	ARB07964	|Homo sapiens|Honduras|07/06/2016		154 NDTG
**46**	ARB07960	|Homo sapiens|Honduras|10/06/2016		154 NDTG
**47**	ARB07931	|Homo sapiens|Jamaica|13/06/2016		154 NDTG
**48**	AOY08535	|Homo sapiens|DominicanRepublic|14/06/2016 Republic|2016/06/14		154 NDTG
**49**	APB03018	|Homo sapiens|USA|21/06/2016		154 NDTG
**50**	ARB07974	|Homo sapiens|Puerto Rico|26/06/2016		154 NDTG
**51**	ATG29297	|Homo sapiens|Mexico|05/07/2016		154 NDTG
**52**	ATG29280	|Homo sapiens|Mexico|06/07/2016		154 NDTG
**53**	ATG29301	|Homo sapiens|Mexico|07/07/2016		154 NDTG
**54**	ARB07936	|Homo sapiens|Jamaica|10/07/2016		154 NDTG
**55**	ATG29267	|Homo sapiens|Guatemala|21/07/2016	2	154 NDTG
**56**	APO39231	|Homo sapiens|USA|29/07/2016		154 NDTG
**57**	AQX32984	|Homo sapiens|Honduras|26/08/2016		154 NDTG
**58**	APH11519	|Homo sapiens|Singapore|27/08/2016		154 NDTG
**59**	APH11522	|Homo sapiens|Singapore|27/08/2016		154 NDTG
**60**	APH11526	|Homo sapiens|Singapore|27/08/2016		154 NDTG
**61**	APH11536	|Homo sapiens|Singapore|28/08/2016		154 NDTG
**62**	APH11556	|Homo sapiens|Singapore|28/08/2016		154 NDTG
**63**	APH11539	|Homo sapiens|Singapore|29/08/2016		154 NDTG
**64**	APH11534	|Homo sapiens|Singapore|30/08/2016		154 NDTG
**65**	APH11611	|Homo sapiens|Thailand|30/08/2016		154 NDTG
**66**	APH11493	|Homo sapiens|Singapore|04/09/2016		154 NDTG
**67**	APH11502	|Homo sapiens|Singapore|07/09/2016	4	154 NDTG
**68**	APH11511	|Homo sapiens|Singapore|13/09/2016		154 NDTG
**69**	APH11588	|Homo sapiens|Singapore|15/09/2016		154 NDTG
**70**	APO39241	|Homo sapiens|USA|03/10/2016		154 NDTG
**71**	AQX32988	|Homo sapiens|Venezuela|19/10/2016		154 NDTG
**72**	ARK18853	|Homo sapiens|China|01/11/2016	5	154 NDTG
**73**	APO08504	|Homo sapiens|China|03/11/2016		154 NDTG
**74**	BAX00477	|Homo sapiens|Japan|22/11/2016		154 NDTG
**75**	ASU55416	|Homo sapiens|Colombia|12/2016	2	154 NDTG

### Phylogeny

**Figure 1.**
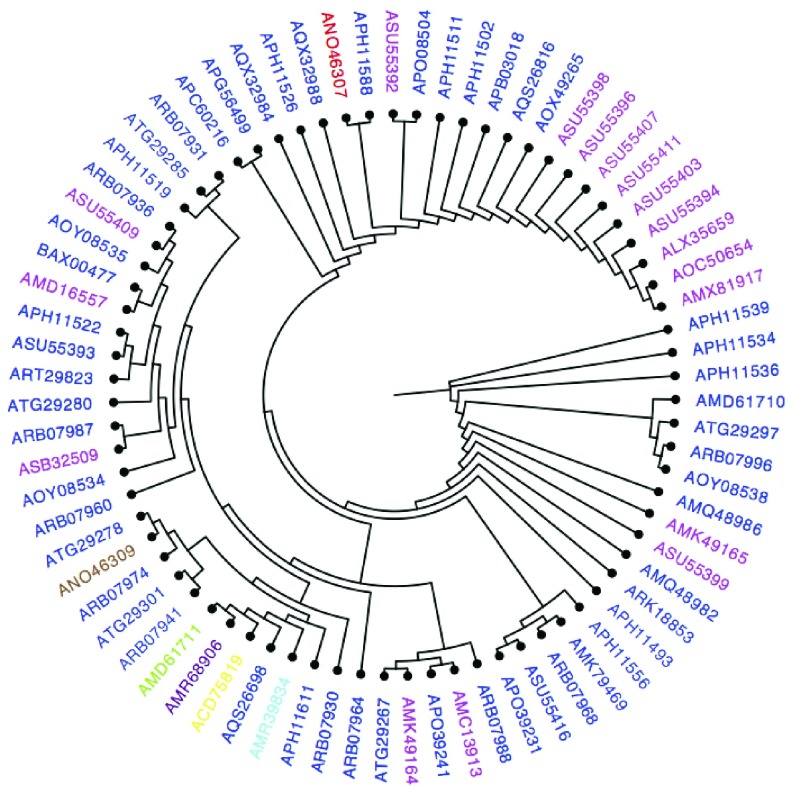
Phylogenic tree of the unique E protein sequences between the years 1968–2017. Year 1968 colored (purple), 2007 (yellow), 2010 (cyan), 2012 (green), 2013(red), 2014 (brown), 2015(magenta), and 2016 (blue).

A phylogenetic tree of unique E protein sequences was constructed using the maximum-likelihood function in PhyML software, with 100 bootstrap replications ( Figure 1). Zika strain accession numbers shown in the phylogenetic tree denote the year of isolation. Notably, E protein sequences from isolates collected in 1968, 2007, 2010, and 2012 clustered in one group, indicating that the associated strains are closely related ( Figure 1). However, sequences from strains isolated in 2013 and 2014 exhibited divergence from the sequences of strains isolated in previous years ( Figure 1).

### Antigenic epitopes

A number of recent reports describe the isolation of mAbs specific for Zika E protein ^23– 28^. These antibodies bind preferentially to epitopes located in DII and DIII of the E glycoprotein ( Table 2). A total of 7 neutralizing mAbs have been isolated from Zika-virus infected humans, and all of these mAbs bind to discontinuous epitopes of E protein. Of note, ZIKV-117 and ZIKV-19 mAbs recognize epitopes located in DII ( Table 2), whereas ZIKV-12 and ZIKV-15 mAbs recognize epitopes located in the FL region, and mAbs ZIKV-Z006, and ZIKV-116 as well as the ZKA 190 mAb recognize epitopes located in DIII. Significant overlap between antigenic epitopes specific for ZIKV-Z006 and ZIKV-116 was observed, as three residues in the epitope recognized by the ZIKV-116 mAb are shared with the epitope recognized by ZIKV-Z006 ( Table 2). In addition, the epitope region recognized by the ZKA 190 mAb overlapped with that of the mAb specific for ZIKV-Z006 in two amino acids residues.

**Table 2.** List of B cell epitopes of Zika E protein mapped with neutralizing antibodies from infected humans and vaccinated mice.

	IEDB ID	Epitope	E Domain	mAb	References
**Human Epitopes**	605975	D67, Q89, K118	DII	ZIKV-117	23
	605976	T309, E393, K394	DIII	ZIKV-116	23
	605977	W101	FL	ZIKV-15	23
	605978	W101, F108	FL	ZIKV-12	23
	605979	W217, F218, D220, P222	DII	ZIKV-19	23
	628722	Y305, S306, L307, T309, A310, A311, G334, T335, D336, K340, Q350, T351, L352, V391, G392, E393, K394, K395	DIII	ZIKV-Z006	24
	733913	Y305, A333, T335, E370, N371	DIII	ZKA 190	25
**Mouse Epitopes** *Discontinuous*	540699	T76, Q77, W101, G102, G104, C105, G106, L107, F108	DII	ZIKV-E- 2A10G6	20
	558355	K301, T315, K316, I317, P318, A319, E320, T321, L322, T327, E329, N362, V364, I365, T366, E367, S372, K373, M374, M375, E377	DIII (C-C’ loop)	ZV-48	26
	558356	L307, K340, P342, A343, Q344, V347, D348, Q350, T351, L352, T353, P354, L358, D384, Y386	DIII (LR)	ZV-54	26
	558357	L307, K340, P342, A343, Q344, V347, D348, Q350, T351, L352, T353, P354, V355, L358, V391	DIII (C-C’ loop)	ZV-64	26
	558358	T309, A310, A311, F312, T313, F314, Q331, Y332, A333, G334, T335, D336, G337, S368, E370, N371, E393, K394, K395, I396, T397	DIII (LR)	ZV-67	26
**Mouse Epitopes** *Linear*	745460	D247, A248, H249, A250, K251, R252, Q253, T254, V255, V256, V257, L258, G259, S260, Q261, E262, G263, A264, V265	DII	NA	27
	745469	G383, D384, S385, Y386, I387, V388, I389, G390, V391, G392, D393, K394	DIII	NA	27
	745482	L300, K301, G302, V303, S304, Y305, S306, L307, C308, T309, A310, A311, F312, T313, F314, T315, K316, V317, P318, A319, E320, T321	DIII	NA	27
	753507	L352, T353, P354, V355, G356, R357, L358, I359, T360, A361, N362, P363, V364, I365, T366, E367	DIII	NA	27
	745501	T427, A428, W429, D430, F431, G432, S433, V434, G435, G436, V437, F438, N439, S440, L441, G442, K443	DIII	NA	27

A total of 10 B-cell epitopes of Zika E protein were found to elicit humoral antibody responses in vaccinated mice ( Table 2). Five of these epitopes were shown to be linear and elicited the production of neutralizing antibodies ( Table 2). An additional five discontinuous epitopes have been characterized based on antibodies obtained from vaccinated mice ( Table 2). The majority of those epitopes are bound to DIII domain of E.

To identify amino acid substitution mutations occurring in B-cell epitopes, we aligned the sequences of 75 unique E protein amino acids sequences among the 422 pre-epidemic and epidemic Zika strains identified. The sequence of Zika/Nigeria/9/9/1968 was used as a reference, and the E protein sequences were mapped against all of the mAbs from Zika infected humans or vaccinated mice. We then annotated the mutations in Zika E glycoprotein at predefined B-cell epitopes. Only nine Zika strains were found to carry mutations in the predefined B-cell epitopes recognized by mAbs from Zika-virus infected humans. Of note, two amino acids substitutions (R335T) and (D393E) appeared in 2007 ( Figure 2A). These amino acids substitutions were retained in all subsequent strains from 2007 to 2016. Interestingly, no unique mutations were observed in predefined B-cell epitopes for isolates collected between 2008 and 2014. However, in 2015, two additional substitutions of alanine residues for threonine residues appeared at amino acid positions 309 and 333 ( Figure 2A). These mutations were not retained in subsequently isolated Zika strains, with the sequences quickly reverting to those of previously isolated strains. The greatest number of amino acid substitution mutations occurred in 2016; the majority of the mutations identified in 2016 involved several deletions in specific regions of the predefined B-cell epitopes ( Figure 2A). Of note, the Dominican Republic/6/6/2016 strain exhibited significant deletions in B-cell epitopes recognized by ZIKV-Z006 mAb ( Figure 2A). Surprisingly, 3 B-cell epitopes were completely conserved in the pre-epidemic and epidemic strains and indeed have not changed for nearly 50 years ( Figure 2A). These epitopes are recognized by the mAbs ZIKV-117, ZIKV-15, and ZIKV-19. Thus, as these mAbs could exhibit cross-reactivity against a wide range of Zika strains, they have potential for use in the development of vaccines and immunotherapeutics targeting Zika ( Figure 2A).

**Figure 2A.**
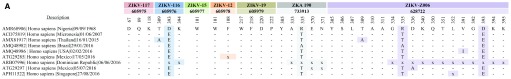
B-cell epitopes alignments mapped with human anti-E monoclonal antibodies. A total of 9 Zika strains are variable at predefined B-cell epitopes during 1968–2017.

Conversely, none of 10 mouse B-cell epitopes were completely conserved among all Zika strains ( Figure 2B, Figure 2C). Of note, the number of Zika strains exhibiting variations in the amino acid sequence at predefined antigenic epitopes of the E protein was higher in mAbs characterized in vaccinated mice than in mAbs characterized in infected humans ( Figure 2B, Figure 2C). In addition, both the linear and discontinuous epitopes of vaccinated mice exhibited variations beginning in 2014 and continuing in subsequent years ( Figure 2B, Figure 2C). The majority of mAbs in vaccinated mice recognized the DIII of E protein, and these epitopes exhibited higher rates of sequence variation than did the mAbs recognizing DII ( Figure 2B, Figure 2C). Of note, the ZIKV-E-2A10G6 mAb binds to a highly conserved discontinuous epitope in which a single amino acid deletion was observed in Zika strain ATG29285| *Homo sapiens*|Mexico|17/05/2016. Remarkably, discontinuous epitopes bound the ZV-67 mAb exhibited the highest degree of variation in amino acid sequence among all the Zika B-cell epitopes examined ( Figure 2B). Sequence variations were also observed in all of the Zika E protein linear epitopes ( Figure 2C).

**Figure 2B.**
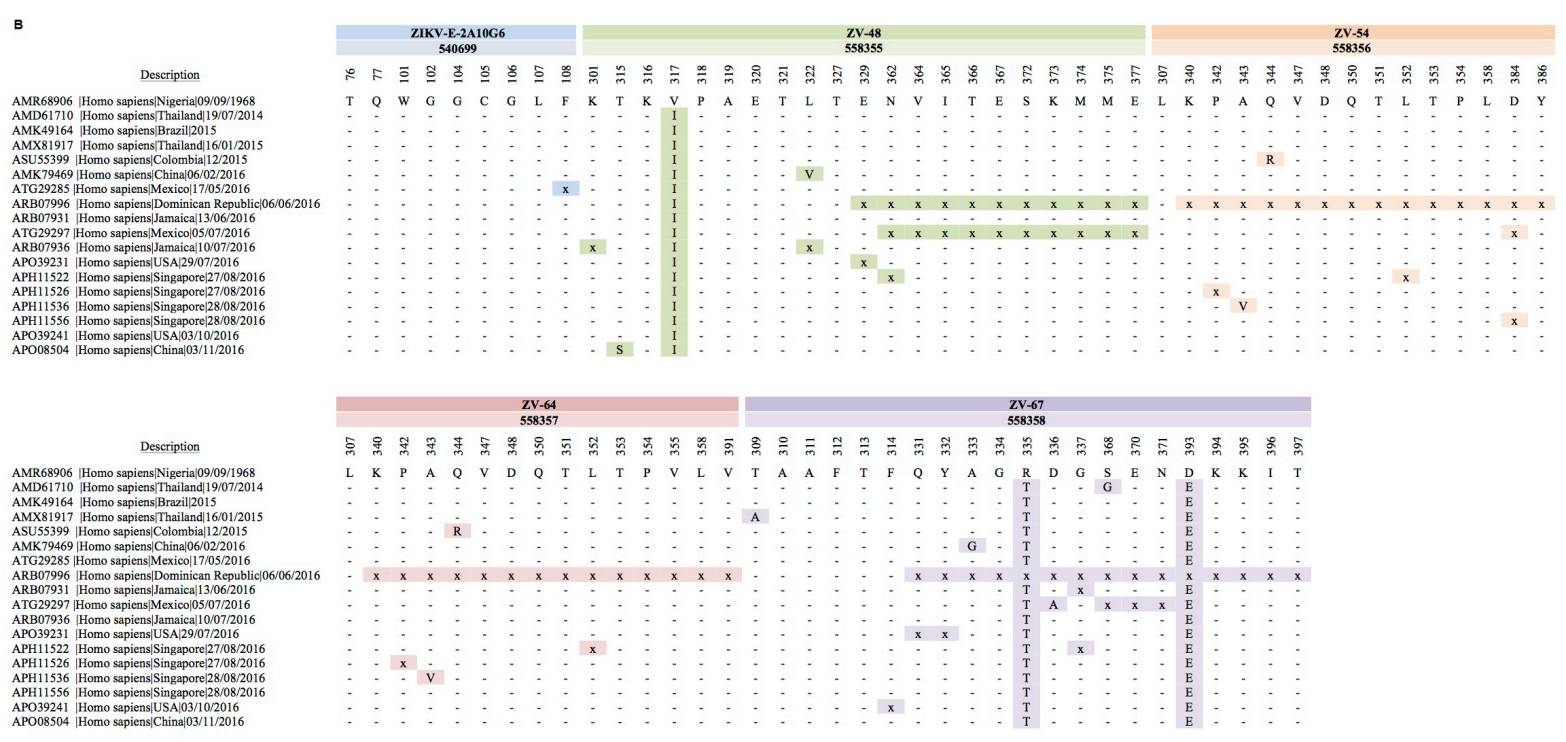
Discontinuous B-cell epitopes alignments mapped with mice anti-E monoclonal antibodies. A total of 18 Zika strains were variable at predefined B-cell epitopes during 1968–2017.

**Figure 2C.**
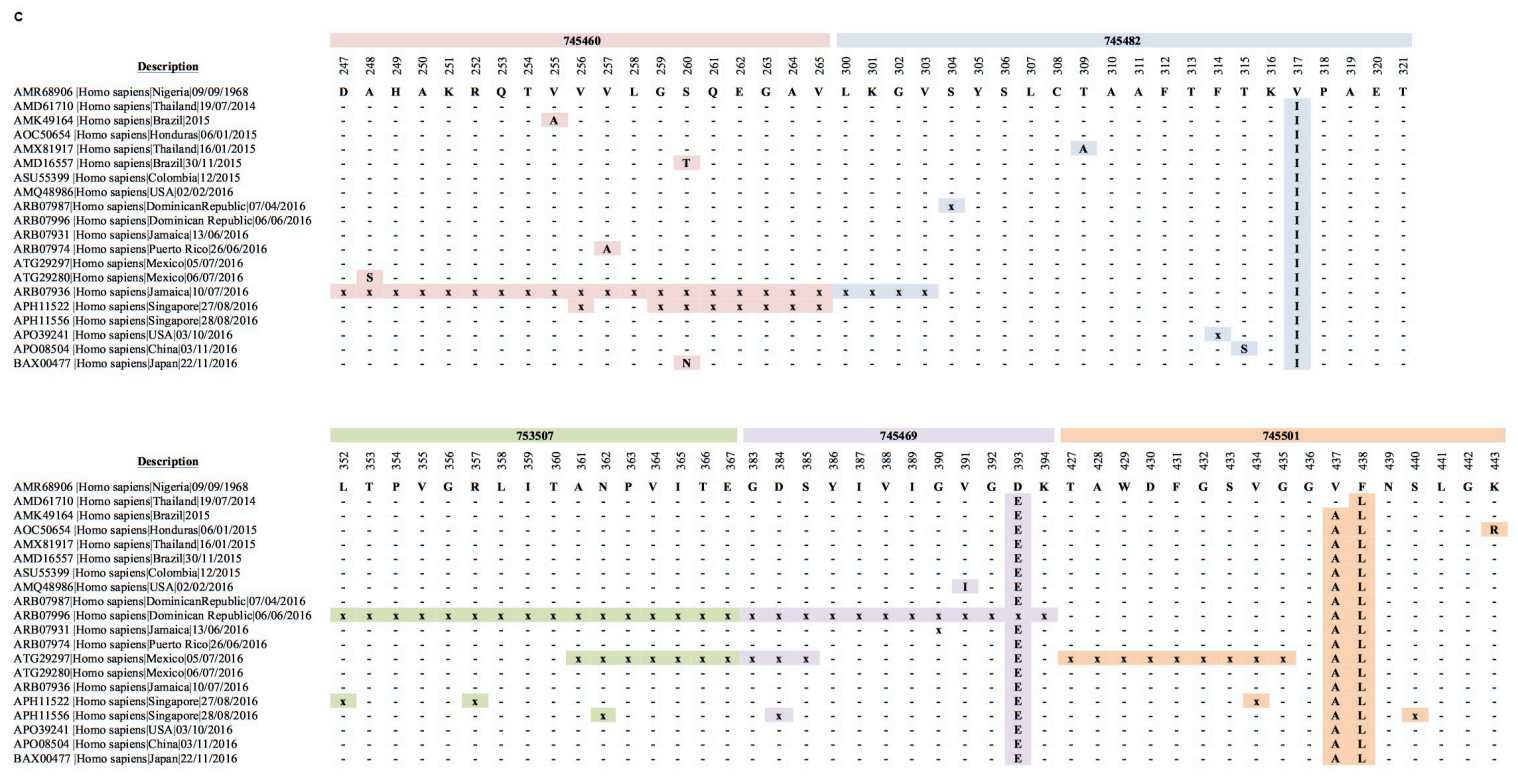
Linear B-cell linear alignments mapped with mice anti-E antibodies. A total of 20 Zika strains were variable at predefined B-cell epitopes during 1968–2017.

### E protein homology modeling

**Figure 3.**
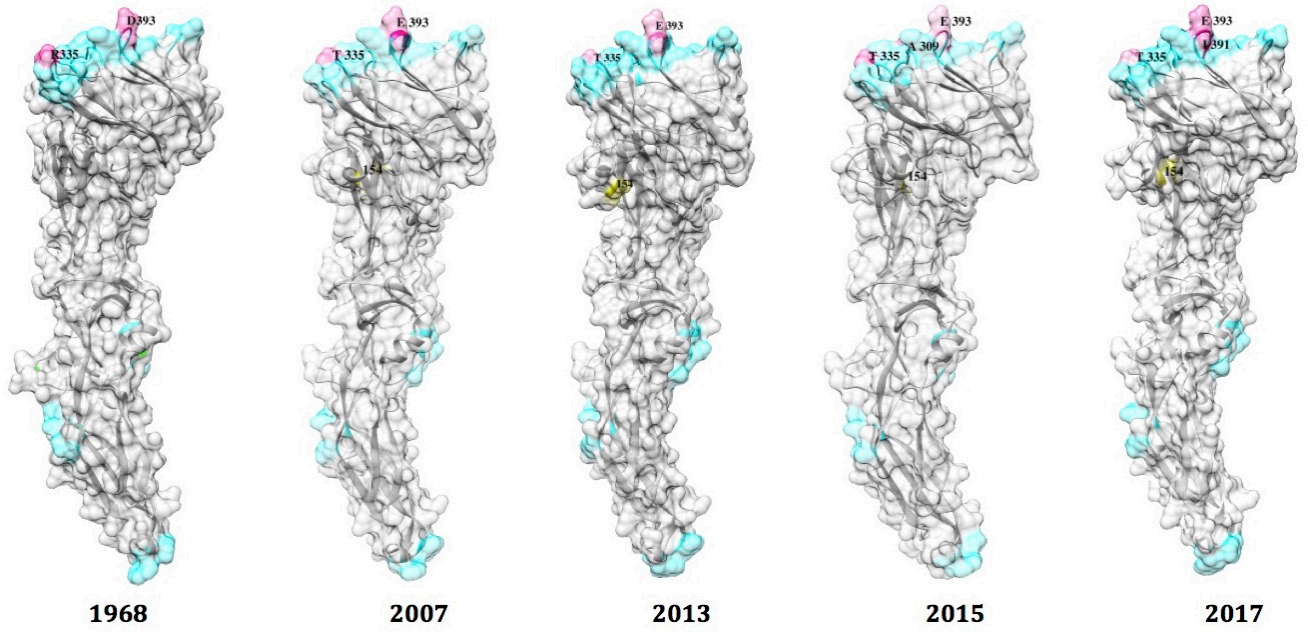
Comparison of B-cell epitopes of monomeric E between the years 1968, 2007, 2010, 2015, and 2016. Homology models of E were built from PDB: 5GZN chain A from strains of following E sequences: AMR68906| Homo sapiens |Nigeria|09/09/1968, ACD75819| Homo sapiens |Micronesia|01/06/2007, ANO46307| Homo sapiens/French Polynesia/11/2013, AMX81917/Homo sapiens/Thailand/16/01/2015, and AMQ48986 | Homo sapiens |USA|02/02/2016. Color blue color represents amino acids conservation at B-cell epitopes and color pink represents amino acids substitutions. Yellow label represent N-glycosylation potential of E.

**Supplementary Figure 1.**
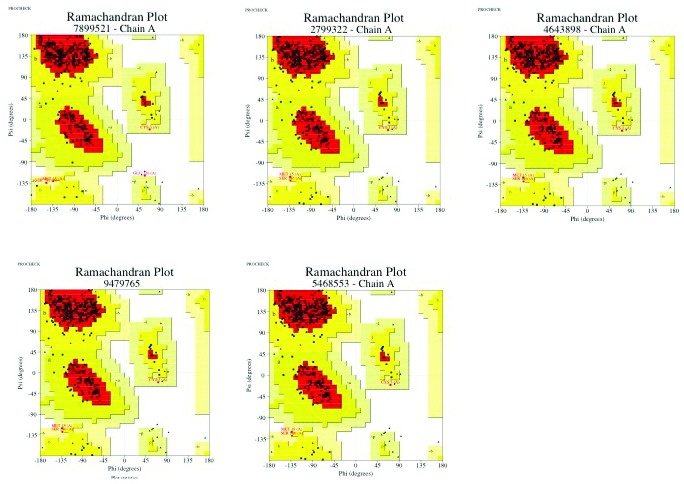
Ramachandran plot analysis of 3D structure of monomeric E. Homology model for sequences of |Nigeria|09/09/1968, ACD75819| Homo sapiens |Micronesia|01/06/2007, ANO46307| Homo sapiens/French Polynesia/11/2013, AMX81917/Homo sapiens/Thailand/16/01/2015, and AMQ48986 | Homo sapiens |USA|02/02/2016 were verified.

The recently solved E protein structure of Zika enabled us to construct a homology model of various E protein sequences. Amino acid substitutions in B-cell epitopes were also annotated on the 3D structure of the E protein. The majority of mutations in the E protein were found to be located within DIII, whereas the B-cell epitopes in DII were highly conserved ( Figure 3). Zika strain *Homo sapiens*/French Polynesia/11/13 did not harbor any additional mutations in B-cell epitopes compared with Zika strain *Homo sapiens*/Micronesia/01/06/07 ( Supplementary Figure 1).

It is known that *N*-glycosylation can mask antigenic epitopes. However, as the glycosylation site in the Zika E protein is located remotely from the predefined B-cell epitopes, glycosylation does not mask the B-cell antigenic epitopes ( Figure 3). This is consistent with reports of glycosylation in E protein of WNV and JEV ^18^. A recent report demonstrated that glycosylation at 154 is critical for Zika infection of both mammalian and mosquito hosts ^17^. Our analyses indicate that antigenic changes occur less frequently in Zika strains ( Figure 3) and suggest that highly effective neutralizing Zika vaccines and immunotherapies for treating infections with known Zika strains are possible. Consequently, monitoring antigenic changes in E proteins over time would be useful for evaluating the cross-neutralizing potential of Zika vaccines against newly mutated strains.

### Mutational stability

Mutation stability was carried out to predict the effects of non-synonymous variants on the stability of E protein and antibody binding. Here, we analyzed the stability of monomeric E protein upon substitutional mutations. Amino acids substitutions can affect the strength, number of interaction, and protein folding and thus increase or decrease binding affinity. To investigate the effect of amino acids substitutions at antigenic epitopes on the stability of E protein and antibody binding of E, 4 prediction tools for mutational stability were selected.

We predicted the stability of B-cell epitope mutations using the 3D structure of Zika E proteins. In both the T309A and T335A substitutions, a polar threonine residue was substituted with a hydrophobic alanine residue. No change in hydrophobicity with the V391I mutation or change in charge with the D393E were observed. In the R335T mutation, a basic residue was substituted with an aromatic residue. Overall, these suggest that defined substitutions in the E glycoprotein are potentially destabilizing. However, these mutations had moderate destabilizing effect, as the ∆∆G values ranged between -0.3 and -0.7 kcal/mol ( Table 3).

**Table 3.** Mutational stability prediction of substituted amino acids at B-cell epitopes of E.

Mutations	mCSM	Effect	SDM	Effect	DUET	Effect	I-Mutant2.0	Effect
T309A	- 0.464 Kcal/mol	Destabilizing	0.12 Kcal/mol	Stabilizing	-0.22 Kcal/mol	Destabilizing	-0.15 Kcal/mol	Decreased stability
T335A	- 0.596 Kcal/mol	Destabilizing	0.12 Kcal/mol	Stabilizing	-0.337 Kcal/mol	Destabilizing	0.33 Kcal/mol	Increased stability
R335T	- 0.304 Kcal/mol	Destabilizing	-0.38 Kcal/mol	Destabilizing	-0.173 Kcal/mol	Destabilizing	NA	NA
V391I	- 0.375 Kcal/mol	Destabilizing	0.08	Stabilizing	-0.022	Destabilizing	-0.25 Kcal/mol	Decreased stability
D393E	- 0.38 Kcal/mol	Destabilizing	0.06 Kcal/mol	Stabilizing	-0.149 Kcal/mol	Destabilizing	NA	NA

## Discussion

Attempts to control the spread of Zika virus via mosquito control have met with limited success. Indeed, within the past 3 years, a Zika pandemic occurred. There is a significant gap in knowledge regarding immunogenic cross-reactivity between Zika strains, even six decades after the first human infection was reported. Bioinformatics approaches can play vital roles in identifying rapidly evolving amino acid residues and thereby facilitate precise mapping of key residues that drive antigenic escape in response to the generation of host neutralizing antibodies. In the present study, we evaluated conserved versus rapidly evolving antigenic regions in predefined B-cell epitopes of the Zika E protein in pre-epidemic and epidemic periods.

The finger-like DII of the Zika E protein contains a FL that is inserted into the endosomal membrane as a result of pH-dependent conformational changes ^14^. The FL is located within the beta-sheet structure in the terminal region of DII and contains a highly conserved hydrophobic peptide that triggers the structural changes required for fusion processes under conditions of low pH ^15^. Our analysis demonstrated that B-cell epitopes in DII of Zika E protein are highly conserved. mAbs ZIKV-117, ZIKV-15, and ZIKV-19 are bound to the highly conserved region of DII and are therefore attractive candidates in the design of Zika vaccines and immunotherapeutics.

The immunoglobulin-like DIII of the Zika E protein contains receptor-binding sites and plays an essential role in attachment and fusion of the virus to host cells ^11, 12^. Importantly, DIII reportedly induces the production of type-specific neutralizing antibodies ^26^, as mAbs isolated from patients infected with either Zika or dengue are highly specific. In the present study, we found that epitopes within E protein DIII vary greatly within Zika strains.

While dengue is considered as a single serotype, it is characterized by four distinct serotypes. The antibody-dependent enhancement (ADE) hypothesis holds that cross-reactive antibodies generated as a result of previous infections with heterologous flaviviruses can enhance the infectivity of other viruses ^42, 43^. Infection with the same serotype elicits a protective immune response, but re-infection with a different serotype can lead to serious disease ^42, 43^. Previous studies demonstrated that mAbs isolated from patients infected with dengue virus cross-react with Zika virus ^44– 47^.

Similarly, mAbs isolated from Zika patients directed against E protein DII cross-react with dengue ^44, 48^, indicating possible increased risk of ADE. Furthermore, another study demonstrated that dengue-virus derived mAbs also cross-react with Zika with high potency ^20^. Those mAbs are bound to quaternary epitopes, which include the site of interaction of E protein dimer with PrM during virus maturation ^20^. In contrast, most mAbs directed against DIII of Zika E protein do not cross-react with dengue ^44^.

In the present study, we compared mAbs of Zika E protein elicited in cases of Zika-virus infected humans versus mAbs induced by Zika vaccination in mice and identified several conserved epitope footprints. The conserved E protein epitopes could be useful in research aimed at developing vaccines that elicit the production of antibodies that provide protection against Zika strains but do not cross-react with dengue. For example, immunization with a peptide cocktail of antigenic DIII epitopes might provide broad protection against a variety of Zika strains yet demonstrate no cross-reactivity with dengue, thus eliminating the possibility of ADE associated with the anti-Zika antibodies.

## Data availability

Zika protein sequences data can be found at the NCBI Zika resource.

Zika B-cell antigenic epitopes can be found at Immune Epitope Database (IEDB).

## References

[1] Kuno G Chang GJ: Full-length sequencing and genomic characterization of Bagaza, Kedougou, and Zika viruses. *Arch Virol.* 2007;152(4):687–696. 10.1007/s00705-006-0903-z 17195954

[2] Lindenbach BD Rice CM: Molecular biology of flaviviruses. *Adv Virus Res.* 2003;59:23–61. 10.1016/S0065-3527(03)59002-9 14696326

[3] Dick GW Kitchen SF Haddow AJ: Zika virus. I. Isolations and serological specificity. *Trans R Soc Trop Med Hyg.* 1952;46(5):509–520. 10.1016/0035-9203(52)90042-4 12995440

[4] Macnamara FN: Zika virus: a report on three cases of human infection during an epidemic of jaundice in Nigeria. *Trans R Soc Trop Med Hyg.* 1954;48(2):139–145. 10.1016/0035-9203(54)90006-1 13157159

[5] Duffy MR Chen TH Hancock WT: Zika virus outbreak on Yap Island, Federated States of Micronesia. *N Engl J Med.* 2009;360(24):2536–2543. 10.1056/NEJMoa0805715 19516034

[6] Cao-Lormeau VM Roche C Teissier A: Zika virus, French polynesia, South pacific, 2013. *Emerg Infect Dis.* 2014;20(6):1085–6. 10.3201/eid2006.140138 24856001 PMC4036769

[7] Zanluca C Melo VC Mosimann AL: First report of autochthonous transmission of Zika virus in Brazil. *Mem Inst Oswaldo Cruz.* 2015;110(4):569–572. 10.1590/0074-02760150192 26061233 PMC4501423

[8] Metsky HC Matranga CB Wohl S: Zika virus evolution and spread in the Americas. *Nature.* 2017;546(7658):411–415. 10.1038/nature22402 28538734 PMC5563848

[9] Apte-Sengupta S Sirohi D Kuhn RJ: Coupling of replication and assembly in flaviviruses. *Curr Opin Virol.* 2014;9:134–142. 10.1016/j.coviro.2014.09.020 25462445 PMC4268268

[10] Zhang Y Corver J Chipman PR: Structures of immature flavivirus particles. *EMBO J.* 2003;22(11):2604–2613. 10.1093/emboj/cdg270 12773377 PMC156766

[11] Stadler K Allison SL Schalich J: Proteolytic activation of tick-borne encephalitis virus by furin. *J Virol.* 1997;71(11):8475–8481. 9343204 10.1128/jvi.71.11.8475-8481.1997 PMC192310

[12] Yu IM Zhang W Holdaway HA: Structure of the immature dengue virus at low pH primes proteolytic maturation. *Science.* 2008;319(5871):1834–1837. 10.1126/science.1153264 18369148

[13] Dai L Song J Lu X: Structures of the Zika Virus Envelope Protein and Its Complex with a Flavivirus Broadly Protective Antibody. *Cell Host Microbe.* 2016;19(5):696–704. 10.1016/j.chom.2016.04.013 27158114

[14] Modis Y Ogata S Clements D: Structure of the dengue virus envelope protein after membrane fusion. *Nature.* 2004;427(6972):313–9. 10.1038/nature02165 14737159

[15] Rey FA Heinz FX Mandl C: The envelope glycoprotein from tick-borne encephalitis virus at 2 A resolution. *Nature.* 1995;375(6529):291–8. 10.1038/375291a0 7753193

[16] Kostyuchenko VA Lim EX Zhang S: Structure of the thermally stable Zika virus. *Nature.* 2016;533(7603):425–8. 10.1038/nature17994 27093288

[17] Bressanelli S Stiasny K Allison SL: Structure of a flavivirus envelope glycoprotein in its low-pH-induced membrane fusion conformation. *EMBO J.* 2004;23(4):728–738. 10.1038/sj.emboj.7600064 14963486 PMC380989

[18] Vaughan AT Roghanian A Cragg MS: B cells--masters of the immunoverse. *Int J Biochem Cell Biol.* 2011;43(3):280–285. 10.1016/j.biocel.2010.12.005 21147251

[19] Heinz FX Auer G Stiasny K: The interactions of the flavivirus envelope proteins: implications for virus entry and release. *Arch Virol Suppl.* 1994;9:339–348. 10.1007/978-3-7091-9326-6_34 7913359

[20] Barba-Spaeth G Dejnirattisai W Rouvinski A: Structural basis of potent Zika-dengue virus antibody cross-neutralization. *Nature.* 2016;536(7614):48–53. 10.1038/nature18938 27338953

[21] Paul LM Carlin ER Jenkins MM: Dengue virus antibodies enhance Zika virus infection. *Clin Transl Immunology.* 2016;5(12):e117. 10.1038/cti.2016.72 28090318 PMC5192063

[22] Xu X Vaughan K Weiskopf D: Identifying Candidate Targets of Immune Responses in Zika Virus Based on Homology to Epitopes in Other Flavivirus Species. *PLoS Curr.* 2016;8: pii: ecurrents.outbreaks.9aa2e1fb61b0f632f58a098773008c4b. 10.1371/currents.outbreaks.9aa2e1fb61b0f632f58a098773008c4b 28018746 PMC5145810

[23] Sapparapu G Fernandez E Kose N: Neutralizing human antibodies prevent Zika virus replication and fetal disease in mice. *Nature.* 2016;540(7633):443–447. 10.1038/nature20564 27819683 PMC5583716

[24] Robbiani DF Bozzacco L Keeffe JR: Recurrent Potent Human Neutralizing Antibodies to Zika Virus in Brazil and Mexico. *Cell.* 2017;169(4):597–609.e11. 10.1016/j.cell.2017.04.024 28475892 PMC5492969

[25] Wang J Bardelli M Espinosa DA: A Human Bi-specific Antibody against Zika Virus with High Therapeutic Potential. *Cell.* 2017;171(1):229–241.e15. 10.1016/j.cell.2017.09.002 28938115 PMC5673489

[26] Zhao H Fernandez E Dowd KA: Structural Basis of Zika Virus-Specific Antibody Protection. *Cell.* 2016;166(4):1016–1027. 10.1016/j.cell.2016.07.020 27475895 PMC4983199

[27] Basu R Zhai L Contreras A: Immunization with phage virus-like particles displaying Zika virus potential B-cell epitopes neutralizes Zika virus infection of monkey kidney cells. *Vaccine.* 2018;36(10):1256–1264. 10.1016/j.vaccine.2018.01.056 29395533

[28] Hatcher EL Zhdanov SA Bao Y: Virus Variation Resource - improved response to emergent viral outbreaks. *Nucleic Acids Res.* 2017;45(D1):D482–D490. 10.1093/nar/gkw1065 27899678 PMC5210549

[29] Kearse M Moir R Wilson A: Geneious Basic: an integrated and extendable desktop software platform for the organization and analysis of sequence data. *Bioinformatics.* 2012;28(12):1647–1649. 10.1093/bioinformatics/bts199 22543367 PMC3371832

[30] Guindon S Dufayard JF Lefort V: New algorithms and methods to estimate maximum-likelihood phylogenies: assessing the performance of PhyML 3.0. *Syst Biol.* 2010;59(3):307–321. 10.1093/sysbio/syq010 20525638

[31] Biasini M Bienert S Waterhouse A: SWISS-MODEL: modelling protein tertiary and quaternary structure using evolutionary information. *Nucleic Acids Res.* 2014;42(Web Server issue):W252–W258. 10.1093/nar/gku340 24782522 PMC4086089

[32] Laskowski RA MacArthur MW Moss DS: *Procheck* - A Program to Check the Stereochemical Quality of Protein Structures. *J App Cryst.* 1993;26:283–291. 10.1107/S0021889892009944

[33] Chen VB Arendall WB 3rd Headd JJ: MolProbity: all-atom structure validation for macromolecular crystallography. *Acta Crystallogr D Biol Crystallogr.* 2010;66(Pt 1):12–21. 10.1107/S0907444909042073 20057044 PMC2803126

[34] Xu D Zhang Y: Improving the physical realism and structural accuracy of protein models by a two-step atomic-level energy minimization. *Biophys J.* 2011;101(10):2525–2534. 10.1016/j.bpj.2011.10.024 22098752 PMC3218324

[35] Vita R Overton JA Greenbaum JA: The immune epitope database (IEDB) 3.0. *Nucleic Acids Res.* 2015;43(Database issue):D405–D412. 10.1093/nar/gku938 25300482 PMC4384014

[36] Pettersen EF Goddard TD Huang CC: UCSF Chimera--a visualization system for exploratory research and analysis. *J Comput Chem.* 2004;25(13):1605–1612. 10.1002/jcc.20084 15264254

[37] Gupta R Jung E Brunak S: Prediction of N-Glycosylation Sites in Human Proteins.2004;46:203–206.

[38] Pires DE Ascher DB Blundell TL: mCSM: predicting the effects of mutations in proteins using graph-based signatures. *Bioinformatics.* 2014;30(3):335–342. 10.1093/bioinformatics/btt691 24281696 PMC3904523

[39] Worth CL Preissner R Blundell TL: SDM--a server for predicting effects of mutations on protein stability and malfunction. *Nucleic Acids Res.* 2011;39(Web Server issue):W215–W222. 10.1093/nar/gkr363 21593128 PMC3125769

[40] Pires DE Ascher DB Blundell TL: DUET: a server for predicting effects of mutations on protein stability using an integrated computational approach. *Nucleic Acids Res.* 2014;42(Web Server issue):W314–W319. 10.1093/nar/gku411 24829462 PMC4086143

[41] Capriotti E Fariselli P Casadio R: I-Mutant2.0: predicting stability changes upon mutation from the protein sequence or structure. *Nucleic Acids Res.* 2005;33(Web Server issue):W306–W310. 10.1093/nar/gki375 15980478 PMC1160136

[42] Kliks SC Nisalak A Brandt WE: Antibody-dependent enhancement of dengue virus growth in human monocytes as a risk factor for dengue hemorrhagic fever. *Am J Trop Med Hyg.* 1989;40(4):444–451. 10.4269/ajtmh.1989.40.444 2712199

[43] Lobigs M Diamond MS: Feasibility of cross-protective vaccination against flaviviruses of the Japanese encephalitis serocomplex. *Expert Rev Vaccines.* 2012;11(2):177–187. 10.1586/erv.11.180 22309667 PMC3337329

[44] Stettler K Beltramello M Espinosa DA: Specificity, cross-reactivity, and function of antibodies elicited by Zika virus infection. *Science.* 2016;353(6301):823–826. 10.1126/science.aaf8505 27417494

[45] Priyamvada L Quicke KM Hudson WH: Human antibody responses after dengue virus infection are highly cross-reactive to Zika virus. *Proc Natl Acad Sci U S A.* 2016;113(28):7852–7857. 10.1073/pnas.1607931113 27354515 PMC4948328

[46] Swanstrom JA Plante JA Plante KS: Dengue Virus Envelope Dimer Epitope Monoclonal Antibodies Isolated from Dengue Patients Are Protective against Zika Virus. *mBio.* 2016;7(4): pii: e01123-16. 10.1128/mBio.01123-16 27435464 PMC4958264

[47] Dejnirattisai W Supasa P Wongwiwat W: Dengue virus sero-cross-reactivity drives antibody-dependent enhancement of infection with zika virus. *Nat Immunol.* 2016;17(9):1102–8. 10.1038/ni.3515 27339099 PMC4994874

[48] Kawiecki AB Christofferson RC: Zika Virus-Induced Antibody Response Enhances Dengue Virus Serotype 2 Replication *In Vitro*. *J Infect Dis.* 2016;214(9):1357–1360. 10.1093/infdis/jiw377 27521359

