# Supplementary material for: Large-scale analysis of B-cell epitopes of envelope: Implications for Zika vaccine and immunotherapeutic development
Source: F1000Res. 2019 Jun 27;7:1624. Originally published 2018 Oct 10. [Version 2] doi: 10.12688/f1000research.16454.2 (PMC6611143; doi:10.12688/f1000research.16454.2)
Supplement: Peer review history

# Peer review: Reviewer response for version 2

**DOI:** 10.5256/f1000research.21556.r50488

**Published:** 2019-07-01

**Authors:** Meirong Jia

**Affiliations:** 1 Roy J. Carver Department of Biochemistry, Biophysics & Molecular Biology, Iowa State University, Ames, IA, USA; 2 University of California, Department of Plant Biology, Davis, CA, USA

Good for indexing.

I confirm that I have read this submission and believe that I have an appropriate level of expertise to confirm that it is of an acceptable scientific standard.

# Peer review: Reviewer response for version 1

**DOI:** 10.5256/f1000research.17985.r42684

**Published:** 2019-05-21

**Authors:** Sunil K Joshi

**Affiliations:** 1 Department of Pediatrics, Miller School of Medicine, University of Miami, Miami, FL, USA; 2 Department of Microbiology & Immunology, University of Miami, Miami, FL, USA

Authors described *in-silico* methods to delineate the data which are particularly important due to high mutation rates by RNA viruses which can generate mutations, that enable them to evade the host immune system. Authors emphasized the structural stability of E protein, which is a key factor for antibody binding. The bioinformatic analysis of B cell epitopes of Zika  is very important study in terms of developing effective vaccine and may be also in cross-protection against other similar viruses.

 In the present study, the authors extracted all of the available E protein sequences for Zika isolates obtained from 1968 to 2017 and constructed three-dimensional (3D) structures of E proteins from various Zika strains using homology modelling. Further the authors investigated the patterns and conservation of E protein B-cell epitopes and assessed their structural stability upon mutation. The authors did meticulous data analysis and discussed very thoroughly.

I confirm that I have read this submission and believe that I have an appropriate level of expertise to confirm that it is of an acceptable scientific standard.

# Peer review: Reviewer response for version 1

**DOI:** 10.5256/f1000research.17985.r39301

**Published:** 2018-11-06

**Authors:** Meirong Jia

**Affiliations:** 1 Roy J. Carver Department of Biochemistry, Biophysics & Molecular Biology, Iowa State University, Ames, IA, USA; 2 University of California, Department of Plant Biology, Davis, CA, USA

Summary:  Zika virus is still a big threat to human health, and E protein is its major structural determinant of immunity. The identification of B-cell epitopes of Zika E protein is crucial for the development of vaccines and immunotherapeutics. Here, the authors conducted systematic bioinformatics analyses of Zika strains isolated between 1968 and 2017, they’ve found conservative epitopes between Zika strains, which could be attractive targets for treating against diverse Zika strains. The research report is generally sound and the finding is scientifically valuable, which would provide guidance for later research in Zika vaccine development. Thus, I would recommend the indexing of this work in F1000Research.

 The followings are some suggestions and comments that might help to make the report more suitable for indexing:

1. In the ‘Introduction’ part, for the 2 ^nd^ paragraph, “After maturation, pr is released from the host cell” is that “PrM” being released instead?
2. In ‘Table 2’, the first row, should “mAB” be “mAb”?
3. In the ‘Abstract’, for the ‘Results’ part, “Our analyses indicate that epitopes recognized by human mAbsZIKV-117, ZIKV-15, and ZIKV-119 were highly conserved”. Should be “ZIKV-19”, please correct it. Also, since there is mutation as shown in ‘Fig 2B’ other than the strict conservation pattern in ‘Fig 2A’, please rewrite the claim that “ZIKV-E-2A10G6 mAb derived from immunized mice was highly conserved across Zika strains”.
4. In the ‘Results’, for E protein homology modelling, the 2 ^nd^ paragraph, “Glycosylation does not mask the B-cell epitope epitopes” should be “antigenic epitopes?”
5. In the ‘Method’ part, for ‘Mapping of antigenic epitopes’, “Potential N-glycosylation sites were defined by the sequence Asp/X/Ser/Thr, where X represents any amino acid except Pro. A threshold of >0.5 suggested an N-glycosylated residue.” Please further explain what values >0.5 would suggest an N-glycosylated residue.
6. The authors described in the ‘Methods’ that “The I-Mutant2.0 tool enables prediction of free energy changes under differing conditions of pH, temperature, neighboring residues, and solvent accessibility.” For the data shown in ‘Table 3’, what conditions did authors apply to conduct the prediction?
7. In the ‘Results’, the last paragraph, “Overall, these suggest that defined substitutions in the E glycoprotein are potentially destabilizing.” Could the authors further discuss what the consequence or effects of destabilization has on virus infection since authors said that the structural stability of E protein is a key factor for antibody binding.
8. In the ‘Discussion’ part, the authors claimed that “The conserved E protein epitopes could be useful in research aimed at developing vaccines that elicit the production of antibodies that provide protection against Zika strains but do not cross-react with dengue.” Yet from the manuscript, it seems that no conserved epitopes in DIII has been identified. If so, authors might need to rewrite the last part of the discussion.

I confirm that I have read this submission and believe that I have an appropriate level of expertise to confirm that it is of an acceptable scientific standard, however I have significant reservations, as outlined above.

# Response

**Published:** 2019-06-11

**Authors:** Iman Almansour

**Affiliations:** Imam Abdulrahman Bin Faisal University, Saudi Arabia

We thank Dr.Jia for the careful reading of the manuscript and the constructive remarks.

 Response to general comments:

 1.Corrections has been made.

 2.Corrections has been made.

 3.Yes, thank you for pointing this out. Corrections were made.

 4.Yes, we are sorry for the unintended mistake. Corrections were made.

 5. Explanation has been made.

 6.pH 7.0 and temperature at 25 C were applied. The details were added in the method section.

 7.In the revised document, we further explained the effect of mutations on protein stability and protein binding (Results section, mutation stability, first paragraph).

 8.In the discussion section, we have explained that immunization with a cocktail of antigenic DIII epitopes might provide broad protection against a variety of Zika strains.
